# Staged thoracoscopic internal traction approach for early repair of long-gap esophageal atresia (LGEA) with distal tracheoesophageal fistula (TEF)

**DOI:** 10.1007/s00383-025-05973-4

**Published:** 2025-01-23

**Authors:** Nitin Sajankila, Cecilia Gigena, Darling Zamorano, Marcela Santos, Alicia Gómez, Isidora Lavado, Anthony L. DeRoss, Manuel Lopez, Maximiliano Maricic, Miguel Guelfand

**Affiliations:** 1 Department of Pediatric Surgery, Cleveland Clinic Children’s Hospital, 8950 Euclid Avenue, Mail Code R3, Cleveland, OH 44106 USA; 2 Department of Pediatric Surgery, Hospital Dr. Exequiel González Cortés, Gran Av. José Miguel Carrera 3300, San Miguel, Región Metropolitana, 8900000 Santiago, Chile; 3 Department of Pediatric Surgery, Hospital Universitario, 12 de Octubre, Av. de Córdoba, 28041 Madrid, Spain; 4 Department Pediatric Surgery, Hospital Universitario Vall d’Hebron, Vall d’Hebron Barcelona Hospital Campus, Passeig de la Vall d’Hebron, 119, Horta-Guinardó, 08035 Barcelona, Spain; 5 Department of Pediatric Surgery, Hospital General De Niños Pedro de Elizalde, Av. Montes de Oca 40, C1270 Buenos Aires, Argentina

**Keywords:** Esophageal Atresia, Long Gap, Internal traction, Tracheoesophageal fistula, Neonatal surgery, Pediatric surgery, Minimally invasive surgery, Thoracoscopic surgery

## Abstract

**Background:**

Long-gap esophageal atresia (LGEA) can complicate the management of esophageal atresia (EA) with or without a tracheoesophageal fistula (TEF). This series describes a short interval, staged, thoracoscopic internal traction approach for LGEA with distal TEF to manage complex anastomotic tension or an anatomically impossible esophageal anastomosis.

**Methods:**

A retrospective review (2018–2024) was performed across four tertiary centers to identify patients with LGEA and distal TEF, managed with a staged, thoracoscopic internal traction approach. In the first stage, the TEF was ligated and internal traction sutures were placed between esophageal pouches. In the second stage, patients underwent primary anastomosis. Short and long-term complications and outcomes were measured.

**Results:**

In total, seven patients were treated with this approach. Gestational ages ranged from 33 to 39 weeks. The average age at the initial surgery was 2.3 days, and the average age at definitive anastomosis was 15.9 days. There were no cases of leak or esophageal dehiscence. Gastroesophageal reflux was a common post-operative complication, occurring in 85.7% of patients.

**Conclusions:**

Temporary internal traction sutures allow for a minimally invasive, safe repair of LGEA with distal TEF, under decreased tension. This technique reduces operative time, and potential complications associated with other long-gap anastomotic techniques.

**Level of evidence:**

IV.

## Background

Long-gap esophageal atresia (LGEA) remains a significant challenge in the surgical management of esophageal atresia (EA). Although a universal definition does not exist, long-gap esophageal atresia can be described as significant tension between the esophageal pouches, prohibiting successful primary anastomosis [1, 2]. In addition, it can be associated with genetic disorders such as Trisomy 21 and VACTERL association, further complicating its care [3, 4]. Although previously thought to be synonymous with EA without a fistula [3, 5], also known as Type I or Type A EA [6], LGEA has been shown to occur in other types of EA as well [3, 4]. Interestingly, a long gap may occur more frequently in EA with a distal fistula, also known as Type III or Type C EA [6], than in EA without the presence of any fistula [7].

Although several different operative strategies have been described for the management of LGEA, historically less consideration has been given to the management of LGEA with a distal fistula, as it was not a well described entity [2, 4, 7]. Operative strategies for LGEA include delayed primary anastomosis, lengthening of the esophagus through myotomy, staged extra-thoracic esophageal advancement (Kimura technique), dynamic internal traction-induced esophageal elongation (Foker technique), and esophageal replacement using conduits such as the stomach, colon, or small bowel [3]. More recently, thoracoscopic approaches for LGEA have also been developed, employing either an external [8] or internal traction (passive or static) approach [9].

We recently showed that a thoracoscopic internal traction approach can be successfully adapted for the management LGEA with a distal fistula [2]. The purpose of the present study was to build on our prior case series, describe our operative technique in more detail, and study both the short and long-term outcomes of our approach.

## Methods

### Patient population

A retrospective analysis was performed at four major tertiary institutions: Cleveland Clinic Children’s Hospital (Cleveland, OH, USA), Hospital Dr. Exequiel González Cortés (San Miguel, Santiago, Chile), Hospital Universitario 12 de Octubre (Madrid, Spain), Hospital Universitario Vall d’Hebron (Barcelona, Spain), and Hospital General de Niños Pedro de Elizalde (Ciudad Autónoma de Buenos Aires, Argentina). All patients that underwent a thoracoscopic repair for LGEA with a distal TEF, between the years 2018 and 2024, were included.

### Surgical technique

Our thoracoscopic approach to LGEA with a distal TEF involved a staged approach with a short passive or static interval of internal traction.

*First stage (internal traction):* Prior to the repair, all patients underwent a rigid bronchoscopy to evaluate the trachea, confirm the presence and the level of a distal fistula, and rule out the presence of any proximal fistula. The patient was positioned in a semi-prone position, with the right side slightly up. Three trocars were placed: 1) a 4 or 5 mm trocar just inferior to the tip of the right scapula, in approximately the fourth or fifth intercostal space (for the thoracoscope), 2) a 5 mm trocar in the right axilla, in the second or third intercostal space (working port), and 3) a 3 mm trocar, posteriorly, in the sixth or seventh intercostal space (additional working port).

The distal esophageal pouch was mobilized down to the level of the diaphragm, taking note of the course of the vagus nerve. The TEF was then identified in relationship to the airway and two 5 mm clips, or looping and transfixing non-absorbable stitches, were placed in the most proximal portion of the fistula, without narrowing the airway. A 10-French straight catheter was passed through the oropharynx, into the esophagus, to aid in the identification of the proximal esophageal pouch in the retropleural space. Then, the proximal esophageal pouch was mobilized up to the level of the neck, preserving the vagus nerve, the azygous vein and taking care not to damage the laryngeal nerves. A test for tension was performed, by approximating the fully mobilized esophageal pouches. If it was anatomically impossible to perform the anastomosis or deemed to be unsafe due to high tension, we proceeded to a staged approach using internal traction.

Two additional 5 mm metal clips were placed on the distal esophageal pouch, and the tracheoesophageal fistula was ligated between the two sets of metal clips, or clips were placed at the opening of the distal pouch if this was already divided. A single 5 mm clip was also placed on the distal aspect of the proximal esophageal pouch to serve as a bolster for the approximating suture. Two full thickness sliding knots, using 4–0 polypropylene or braided Polyester, were placed to approximate both esophageal pouches as much as possible without damaging the esophageal tissue. A 12-French chest tube was placed and an 8-French Replogle tube was advanced into the proximal esophageal pouch to ensure it remained decompressed. No gastrostomy tube (G-tube) was placed in any of the patients in this study. The patient was then returned to the NICU, intubated, sedated, and paralyzed for the remainder of the traction period.

*Second stage (delayed anastomosis):* In the second stage of repair, after a short interval of traction, roughly 4–6 days, trocars were re-inserted through the previous incisions. The esophageal pouches were identified, freed with blunt dissection, and again tested to see if they could be approximated without significant tension, prior to opening them. If necessary, the anastomosis could be aborted and the internal traction step repeated, as an additional stage of repair. Otherwise, we proceeded to perform the anastomosis primarily, over a 6-French Replogle tube, using 5–0 or 6–0 PDS^®^ or Prolene™ sutures. A new 10-French chest drain tube was placed and the procedure was completed.

### Data collection

Demographic and patient characteristic data including sex, gestational age, birthweight and comorbidities were collected. Perioperative data including the number of surgeries or stages of repair prior to anastomosis, the age at each surgery, the age at anastomosis, the total days of traction, timing of the esophagram, and time to initiation of enteral feeds were reported as a mean ± standard deviation (SD). Long-term follow up included the duration of follow up, the presence and management of any esophageal stenosis, and the presence and management of any gastroesophageal reflux disease (GERD).

## Results

Between the years 2018 and 2022, seven patients underwent a thoracoscopic repair of their Type III/C LGEA using a staged approach with a short interval of internal traction. The gestational age of our patients ranged from 33 to 39 weeks, and three of the seven patients studied were female. The average birth weight was 1997.1 $\pm$ 626.4 g and the comorbidities for each patient are displayed in Table 1. No patient had VACTERL or Trisomy 21.

**Table 1:** Patient characteristics

Patient	1	2	3	4	5	6	7
Sex (M/F)	F	F	M	F	M	F	F
Gestational age (weeks)	36	39	36	33	33	34	33
Birth weight (grams)	2850	3070	1860	1500	1400	1600	1700
Comorbidities	Single umbilical artery, patent ductus arteriosus, anemia	Tracheomalacia	Hyperbilirubinemia, pulmonary valve stenosis, patent ductus arteriosus, mild subglottic stenosis, mild tracheomalacia	MCAD deficiency, dysplastic kidney, renal cyst, apnea of prematurity, patent ductus arteriosus, atrial septal defect, ventricular septal defect	–	Dextrocardia	Apnea of prematurity, patent ductus arteriosus, rib and vertebrae malformation

Most patients underwent a two-staged repair (71.4%). However, two patients (28.6%), Patients 3 and 5, required an additional stage of internal traction prior to final anastomosis due to persistent tension and their final anastomosis was delayed until medically stable. On average the total number of days of traction required prior to anastomosis was $13.6\pm 13.1$ days (range: 4 to 43 days). Of note, there were no cases of leaks that occurred while patients were on traction. The mean age at final anastomosis was $15.9\pm 13.2$ days (range: 5 to 45 days). In patients that did not require a second stage of traction prior to anastomosis (Patients 1, 2, 4, 6, and 7), the mean days of traction was $6.4\pm 2.9$ days and the age at final anastomosis was $8.6\pm 3.6$ days. An esophagram to rule out leak was performed approximately a week after surgery and prior to initiating enteric feeds (see Table 2).

**Table 2:** Perioperative data

Patient	1	2	3	4	5	6	7	Mean $\pm$ SD
Number of surgeries prior to anastomosis	1	1	2	1	2	1	1	$1.3\pm 0.5$
Age at first surgery (days)	3	1	3	1	2	2	4	$2.3\pm 1.0$
Age at second surgery (days)	–	–	9	–	40	–	–	–
Age at anastomosis (days)	15	6	23	5	45	7	10	$15.9\pm 13.2$
Total days of traction	12	5	20	4	43	5	6	$13.6\pm 13.1$
Timing of esophagram (days post anastomosis)	5	5	7	10	20	7	10	$9.1\pm 4.8$
Initiation of enteric feeding after anastomosis (days)	5	7	8	11	1	2	2	$5.1\pm 3.4$

Long-term follow up ranged from 7 months to 6 years (see Table 3). Three of the seven patients (42.9%) developed esophageal stenosis by 6 months of age, requiring a single endoscopic dilation for management. Six patients (85.7%) also developed symptomatic GERD during the study period. Four of these six patients (66.7%) were successfully treated with medical management using proton pump inhibitors (PPI). However, two patients (28.6%) required surgical repair, undergoing Nissen fundoplication. Both are doing well, tolerating enteral feeds.

**Table 3:** Long-term follow-up data

Patient	1	2	3	4	5	6	7
Follow-up	6 years	4 years	2 years, 10 months	7 months	4 years	6 years	2 years, 6 months
*Stenosis*							
Yes/No	Yes	Yes	No	No	Yes	No	No
Age at diagnosis	6 months	6 months	–	–	4 months	–	–
Management	Endoscopic dilation	Endoscopic dilation	–	–	Endoscopic dilation	–	–
*Gastroesophageal reflux*							
Yes/No	No	Yes	Yes	Yes	Yes	Yes	Yes
Age at diagnosis	–	8 months	5 months	2 months	4 months		6 months
Management	–	Proton pump inhibitors	Proton pump inhibitors	Nissen Fundoplication	Proton pump inhibitors	Nissen Fundoplication	Proton pump inhibitors treatment

## Discussion

In this study, we have demonstrated that LGEA with a distal fistula can be successfully repaired by adapting a thoracoscopic internal traction approach. This technique does not require the use of a G-tube, and the final anastomosis can be performed after a short duration of internal traction, as little as 5–6 days. Most patients in this study underwent only two stages of repair, with the first stage being internal traction and the second surgery being delayed anastomosis. However, if it was deemed unsafe to perform the anastomosis, as occurred in two patients, an additional period of internal traction could be performed after adjusting the internal traction suture.

Operative repair of LGEA is classically associated with significant morbidity, including fairly high rates of esophageal leak and stenosis and GERD post-operatively. In a large, meta-analysis looking at outcomes after delayed anastomosis for LGEA, in which 43% (194/451) of patients had pure LGEA and 55.9% (252/451) had LGEA with a distal fistula, anastomotic stricture was observed in 57.0% (155/272) and GERD was observed in 47.9% (131/274) of patients [7]. In our small study, we found that almost half of our patients developed esophageal stenosis, and a majority developed GERD, after a staged, thoracoscopic internal traction approach. The esophageal stenosis in each of the patients was treated successfully with endoscopic balloon dilation by the 6-month mark. Also, the majority of patients with post-operative GERD were treated with a PPI alone; however, two did require a Nissen fundoplication.

In another study looking at 169 patients with EA with a distal TEF that underwent surgical repair, 55 (33%) of patients were found to have a stricture at the one-year mark. In this study population, the use of thoracoscopic surgery was not found to be associated with stricture formation on multivariate logistic regression, but the presence of LGEA and use of PPI (signifying the presence of GERD) were [10] These results suggest that the use of a minimally invasive approach alone does not increase the risk for complication after EA repair. However, it is unclear at present if the same is true for LGEA as well, given that LGEA itself is a risk factor for complications after repair.

Our study builds on prior work looking at the effectiveness in a two-staged, thoracoscopic internal traction approach to treating LGEA [11]. In 2017, *Tainaka *et al*.* showed that it was possible to repair thoracoscopically LGEA in two stages using internal traction; however, in their study only one of the five patients included had a distal TEF. Two patients (40%) had a minor anastomotic leak, one patient (20%) had anastomotic stenosis, and four patients (80%) had GERD. Their technique required the use of a G-tube for enteral feeds with delayed oral intake. The average time to initiation of tube feeds was 10 days and the average time to initiation of oral intake was 45 days [11]. In 2018, *Bogusz *et al*.* subsequently demonstrated that in patients with LGEA and without a distal fistula, this same technique could be performed in the neonatal period, without a G-tube and with initiation of enteral feeds with a week [12]. The elimination of the G-tube, which would have required an additional surgery for closure of the gastrostomy, and the early initiation of enteral feeds during the neonatal period, were significant improvements compared to the previous methodology. In our study, we further adapted this approach to patients with LGEA and a distal TEF, demonstrating this variant of LGEA can also be successfully approached with this technique during the neonatal period.

There are several limitations to our study including that this is a small study of only 7 patients with LGEA with a distal TEF and thus additional study is warranted to validate our findings. However, the long-term follow up presented here adds weight to the durability of this type of thoracoscopic approach to repairing LGEA with a distal TEF. Future areas for study include a larger study to understand the true rates of complications and the duration of time required to achieve full enteral feeds after a staged, thoracoscopic, internal traction approach to LGEA with a distal fistula. Furthermore, additional work is needed to understand how outcomes might differ after repair of LGEA with a distal fistula versus pure LGEA. Additionally, the use of technologies such as indocyanine green (ICG) to verify the perfusion of the esophageal anastomosis [13] may help to make decisions regarding anastomosis or additional time for internal traction and help minimize rates of strictures.

## Conclusion

In this study, we demonstrated that a staged, internal traction approach to repairing LGEA with a distal fistula can be successfully performed thoracoscopically, in two stages, with a short-interval of internal traction, and without the use of any G-tube. The rates of post-operative complications, including leaks, strictures, and GERD, after this minimally invasive approach are encouraging and suggest that the presence of this classically challenging variant of EA, should not discourage the use of thoracoscopy for repair. This approach challenges the current paradigm that all Type III/C esophageal atresia cases must be repaired with a primary anastomosis in a single surgery. Instead, it demonstrates that a staged approach is feasible for "long-gap" Type III/C atresia, allowing for a safer anastomosis with reduced tension, thereby improving surgical outcomes.

## Data Availability

No datasets were generated or analysed during the current study.
